# Attentional dysfunction in persistent postural-perceptual dizziness: a narrative review of mechanistic evidence

**DOI:** 10.3389/fneur.2026.1938453

**Published:** 2026-09-09

**Authors:** Shuo Yang, Xi-Can Liu, Jing Chen

**Affiliations:** Department of Neurology, Zhengzhou Central Hospital Affiliated to Zhengzhou University, Zhengzhou, Henan Province, China

**Keywords:** active inference, attentional dysfunction, multisensory integration, neuro-otology, persistent postural-perceptual dizziness (PPPD), precision weighting, visual dependence

## Abstract

Persistent postural-perceptual dizziness (PPPD) is a common chronic functional neuro-otological disorder characterized by persistent non-spinning dizziness, unsteadiness, and heightened sensitivity to upright posture, motion, and visually complex environments. Several conceptual models have been proposed to explain its persistence, including failure of readaptation after a destabilizing event, maladaptive multisensory integration with excessive visual or somatosensory weighting, and prediction-actual sensory mismatch with amplified prediction errors. This narrative review synthesizes neuroimaging, behavioral, psychophysical, and clinical evidence to examine attentional dysfunction as a candidate organizing mechanism that may link these partly overlapping pathways. Structural and functional alterations involving the dorsolateral prefrontal cortex (DLPFC), anterior cingulate cortex (ACC), posterior insula, precuneus, visual cortex, vestibular cortical regions, and large-scale attention, salience, sensorimotor, visual, and default-mode networks suggest distributed functional reorganization rather than a single lesional substrate. Behavioral studies indicate that postural control and self-motion perception may become excessively attention-dependent in subsets of patients, with dual-task effects, altered visual exploration, stiffened postural strategies, and abnormal sensory-perceptual scaling. Clinical studies further show balance vigilance, attentional bias, and heterogeneous symptom phenotypes, including visual intolerance, quiet standing or sitting intolerance, passive motion intolerance, and active motion intolerance. We propose a revised active-inference model in which attention contributes to dysregulated precision weighting across visual, vestibular, somatosensory, interoceptive, and postural prediction-error signals. This model is hypothesis-generating rather than definitive. It accommodates patients with or without a clear preceding vestibular disorder and patients whose symptoms occur without overt body motion. The framework highlights testable targets for future studies using event-related potentials, computational modeling, dual-task paradigms, and phenotype-stratified treatment trials.

## 1 Introduction

Persistent postural-perceptual dizziness (PPPD) is a chronic functional vestibular disorder characterized by persistent non-spinning dizziness, subjective unsteadiness, and heightened sensitivity to motion, upright posture, and visually complex environments (1). The disorder was formally defined by the Barany Society in 2017, unifying several previously fragmented diagnostic entities including phobic postural vertigo (PPV), chronic subjective dizziness (CSD), space-motion discomfort (SMD), and visual vertigo (VV) (2). According to the Barany Society criteria, diagnosis requires persistent dizziness or unsteadiness lasting 3 months or longer, with symptoms exacerbated by upright posture, active or passive motion, and exposure to complex or moving visual stimuli (1). Routine vestibular function tests and structural neuroimaging are often normal or insufficient to explain the degree of disability, creating a clinical dissociation between subjective symptom burden and conventional objective findings (3).

Epidemiological data indicate that PPPD is one of the most common causes of chronic dizziness in clinical practice. It accounts for approximately 15–20% of dizziness presentations in tertiary care settings and up to 40% in specialized vestibular centers (4). The disorder predominantly affects adults aged 30–50 years, with a marked female predominance, and women are affected approximately four times more frequently than men (5). PPPD is often precipitated by acute vestibular events such as benign paroxysmal positional vertigo (BPPV), vestibular neuritis, or vestibular migraine, but it may also follow neurological or medical illness, psychological distress, prolonged imbalance, or a perceived threat to stability without a clearly documented peripheral vestibular lesion (1, 5). This diversity of triggers argues against a purely peripheral vestibular disorder and points toward central mechanisms of symptom persistence. A recent prospective clinical cohort further supports this view by showing idiopathic onset in a substantial subgroup, marked subjective-objective dissociation, and improvement after structured multimodal therapy (6).

The functional impact of PPPD is substantial. Patients frequently report reduced mobility, avoidance of visually stimulating environments such as supermarkets or crowded spaces, difficulty with walking or passive motion, and impairment in occupational and social functioning (7). Secondary anxiety, hypervigilance toward bodily sensations, and maladaptive avoidance behaviors are common and may further reinforce symptom chronicity (3). The disorder is now recognized in the International Classification of Diseases, 11th Revision (ICD-11) as a functional neuro-otologic disorder, positioning it at the interface between neurology, otolaryngology, psychiatry, psychology, and rehabilitation medicine (8–10).

Despite growing clinical recognition, the neurobiological mechanisms underlying PPPD remain incompletely understood. One influential conceptual account, the failure of readaptation model, describes how acute protective strategies may persist after the original destabilizing event has resolved. However, this model should be regarded as a hypothesis-generating framework rather than a settled consensus theory. It does not fully explain why this transition occurs in some individuals but not others, why symptoms can occur in patients without a clear preceding vestibular disorder, or why some patients report dizziness during sitting or supine conditions in the absence of overt body motion. Recent mechanistic studies have emphasized two partly overlapping pathways for symptom persistence: maladaptive multisensory integration, characterized by abnormal weighting of visual, vestibular, or somatosensory inputs; and prediction-actual sensory mismatch, in which internal models of motion and posture fail to update appropriately, leading to amplified prediction errors and distorted perception of bodily stability (11–13).

A growing body of evidence suggests that attentional dysfunction may represent a cross-domain mechanism linking these pathways. Neuroimaging studies have identified structural and functional alterations in attention-related, salience, vestibular, visual, sensorimotor, and default-mode networks in PPPD patients (14–23). Behavioral and clinical investigations have revealed maladaptive attentional resource allocation, heightened vigilance toward postural and visual stimuli, altered visual exploration during locomotion, and attentional biases toward threat-related cues (5, 11, 24–28). This review aims to synthesize evidence across these domains to evaluate the hypothesis that attentional dysfunction contributes to PPPD pathophysiology. We first outline current conceptual models, then critically examine the evidence implicating attention as an organizing process that may bridge multisensory integration deficits, predictive processing abnormalities, and clinically heterogeneous symptom phenotypes.

## 2 The failure of readaptation model

The failure of readaptation model is a widely used conceptual account of the onset and persistence of PPPD, but it should not be interpreted as a fully validated symptom taxonomy or as the only pathophysiological explanation (3, 5). According to this model, PPPD can develop when protective responses to dizziness, imbalance, or perceived postural threat fail to recede after the precipitating condition has stabilized. Rather than reflecting structural injury to neural tissue, PPPD represents a maladaptive continuation of responses that were adaptive during acute uncertainty but become counterproductive when maintained chronically.

### 2.1 Acute-phase compensatory responses

Acute vestibular disturbances such as vestibular neuritis, BPPV, vestibular migraine, panic attacks with perceived instability, syncope, medical illness, or prolonged imbalance can disrupt balance and spatial orientation (1, 5). In response, patients may increase reliance on visual or somatosensory cues, consciously monitor posture and gait, stiffen the trunk and limbs, and become more sensitive to environmental motion cues (3, 5). These strategies are best understood as a conceptual synthesis supported by converging primary studies rather than as four mandatory features present in every patient.

Several strands of primary evidence support this synthesis. Classical posturographic studies in visual vertigo showed that moving visual environments can provoke dizziness and sway changes in visually dependent patients (29). In PPV, increased body sway in the 3.5–8 Hz band suggested a stiffened strategy with increased anti-gravity muscle co-contraction rather than frank loss of stability (26). Stabilogram diffusion analysis further showed abnormal interaction between open-loop and closed-loop postural control, consistent with excessive conscious control of stance (25). Gait studies demonstrated slower, more cautious walking in PPV, with dual-task and eyes-closed conditions supporting increased attentional control and greater visual reliance during locomotion (27). Oculomotor studies also showed altered visual exploration during walking in PPV, including restricted gaze behavior and reduced horizontal eye-head exploration in complex environments (28). In PPPD itself, the Sensory Organization Test showed impaired postural control across multiple sensory conditions (30), and more recent work has used visual-vestibular stimulation to characterize context-dependent changes in postural stability and perceived egomotion (31, 32).

These findings support the idea that acute and chronic functional dizziness may involve protective but costly strategies. However, they also show why the framework must be applied cautiously. Stiffness, visual reliance, and hypervigilance are not diagnostic requirements, and the dominant provoking stimulus may differ across patients.

### 2.2 Normal recovery and withdrawal of compensatory strategies

In most individuals, as vestibular function stabilizes or central compensation progresses, these high-alert strategies are gradually withdrawn over a period of days to weeks. Automatic postural control re-emerges, sensory weighting becomes more flexible, and internal models of motion, posture, and spatial orientation are updated to reflect the new physiological state. This recovery process is supported by flexible multisensory recalibration and predictive suppression of expected, non-threatening sensory feedback (33, 34).

### 2.3 Maladaptive persistence

In individuals who develop PPPD, this transition back to automatic and context-flexible control may be incomplete. The brain continues to treat normal postural sway, benign environmental motion, passive motion, or internally generated bodily sensations as overly salient. The resulting pattern may include excessive visual or somatosensory reliance, rigid and effortful postural control, heightened monitoring of bodily sensations, and difficulty disengaging from motion cues (3, 7, 11). Risk factors that increase vulnerability include acute anxiety or intense fear during the triggering event, high neuroticism, elevated bodily vigilance, prolonged imbalance, delayed vestibular recovery, and catastrophic interpretations of bodily sensations (5, 11, 24).

Importantly, this model does not require a previous peripheral vestibular disorder in every patient. The Barany Society criteria recognize that PPPD may follow peripheral or central vestibular disorders, other medical illnesses, psychological distress, or chronic conditions that gradually increase dizziness and imbalance (1). Therefore, a “high-alert” state can be initiated not only by objective postural threat, but also by perceived instability, interoceptive threat, migraine-related sensory sensitivity, panic-related bodily alarm, or prolonged uncertainty about bodily orientation. This broader framing is essential for explaining primary PPPD-like presentations and patients whose main symptoms occur during sitting, quiet standing, passive motion, or visually induced egomotion rather than after an acute vestibular insult.

### 2.4 Limitations of the model

The failure of readaptation model provides a useful macro-level account of clinical evolution, but it has important limitations. It describes a persistence of protective strategies without specifying the neural and computational mechanisms that maintain them. It also risks overemphasizing visual dependence and upright postural threat if applied too narrowly. These gaps have motivated closer investigation of multisensory integration, predictive processing, attention, salience, interoception, and phenotype-specific symptom mechanisms in PPPD.

## 3 Two mechanistic pathways converging on attention

Two major mechanistic pathways have been proposed to explain the persistence of PPPD symptoms at the neurobiological level. Critically, both pathways implicate attentional processes, though the nature and extent of attention’s involvement has only recently begun to be systematically examined.

### 3.1 Pathway one: multisensory integration failure and visual dependence

Healthy balance control relies on continuous integration of vestibular, visual, and somatosensory inputs. The brain flexibly adjusts the relative weighting of each sensory channel based on its estimated reliability in a given context, a process known as multisensory re-weighting (34, 35). For example, in a dark or uneven environment where visual signals are unreliable, their influence is down-weighted while vestibular and somatosensory inputs are amplified. In visually rich but stable environments, visual cues may receive greater weight to support spatial orientation.

In PPPD, this dynamic re-weighting process appears to become less flexible. Neuroimaging studies have reported structural and functional alterations in regions involved in vestibular, visual, and multisensory processing, including the posterior insula, parietal operculum, temporoparietal junction (TPJ), parieto-insular vestibular cortex (PIVC), precuneus, visual cortex, sensorimotor cortex, and cerebellum (14, 15, 17–23, 36–39). These findings do not indicate a single damaged structure. Instead, they suggest distributed functional reorganization across vestibular, visual, sensorimotor, salience, and attentional-control networks.

One behavioral consequence of altered sensory weighting is visual dependence. Classical visual vertigo studies showed that moving visual stimuli can provoke dizziness and postural instability in visually dependent patients (29). In PPPD and related functional dizziness conditions, patients often report discomfort in supermarkets, patterned environments, traffic, scrolling screens, or crowded spaces. However, the evidence now suggests a more nuanced picture. Visual dependence is prominent in many patients, but it is not universal. Some patients are mainly provoked by walking, passive motion, quiet standing, or sitting (31, 40–43). Recent visual-vestibular studies further challenge a simple “generalized visual hypersensitivity” account by showing context-dependent effects of body position, vestibular stimulation, and stimulus type on perceived egomotion and postural sway (31, 40).

Attention may contribute to this multisensory imbalance without being reducible to visual dependence. Passamonti et al. showed that neurotic traits in PPPD modulated prefrontal connectivity with visual motion areas during vertical visual motion stimulation (16). Li et al. reported altered intra- and inter-network connectivity, including abnormal coupling between sensorimotor and occipital visual networks and reduced efficiency in the precuneus (18). Yagi et al. found that visual stimulation altered resting-state functional connectivity among vestibulo-visuo-somatosensory and spatial cognitive cortical areas, including increased connectivity from visual areas to spatial cognitive and prefrontal areas after stimulation in PPPD (20). More recent network analyses by Li et al. identified abnormal circuits involving occipital visual cortex, precuneus, sensorimotor cortex, multisensory vestibular cortex, and cerebellum, with altered topology associated with dizziness handicap (19). Together, these studies support a model in which attention can bias relative precision weighting across visual, vestibular, and somatosensory signals, while leaving room for non-visual dominant phenotypes.

### 3.2 Pathway two: prediction-actual sensory mismatch

From a computational neuroscience perspective, balance and postural control can be understood as a process of predictive coding. The brain continuously generates forward models that predict the sensory consequences of its own movements, such as the expected proprioceptive and vestibular feedback from normal body sway. These predictions are compared against actual sensory input, and only unexpected or unpredicted signals (prediction errors) are passed forward for higher-order processing (33, 44). Predicted, expected signals are effectively “canceled out” through a process of predictive suppression, allowing for efficient, automatic postural control that requires minimal conscious attention.

In PPPD, this predictive system appears to operate with abnormal gain and threshold parameters. Castro et al. reviewed evidence from brain imaging, behavioral studies, and perceptual experiments and concluded that PPPD involves a failure to attenuate sensory prediction errors, resulting in minor physiological oscillations, such as those caused by heartbeat, breathing, or normal postural sway, being misinterpreted as significant threats to balance (11).

Recent behavioral and psychophysical studies have provided direct evidence for this predictive processing abnormality (45–47). Storm et al. examined visual and vestibular motion perception in PPPD patients and found a pattern of abnormal sensory-perceptual scaling: patients showed lower galvanic vestibular stimulation thresholds but paradoxically higher visual motion coherence thresholds (46). Notably, patients also demonstrated a relationship between rotatory egomotion perception thresholds and false-alarm perceptions of motion in sham (no-motion) conditions. This finding is consistent with the hypothesis that abnormal precision weighting in predictive coding leads to both heightened sensitivity to some sensory channels and inappropriate false-positive percepts.

Helmchen et al. provided complementary evidence by comparing perceived sway against objectively recorded postural sway (47). PPPD patients demonstrated a significant overestimation of their own body sway relative to actual measured sway, indicating a discrepancy between predicted and actual sensory states, a direct demonstration of prediction error amplification. This perceptual overestimation is particularly important because it reveals that the problem is not simply one of sensory input dysfunction, but rather a higher-order distortion in how sensory signals are interpreted and consciously experienced.

The relevance of attention to this predictive coding abnormality becomes apparent when considering the mechanisms underlying prediction error regulation. In the predictive coding framework, the estimated precision, or gain, of sensory prediction errors is modulated by attention (48). When attention is excessively directed toward bodily sensations or environmental motion, the corresponding prediction errors may be weighted as disproportionately reliable, causing them to override stable top-down predictions about postural stability. Storm et al. explicitly interpreted their findings as reflecting abnormal sensory-perceptual scaling consistent with altered precision weighting (46). Helmchen et al. showed that PPPD patients significantly overestimate their own postural sway relative to objectively measured sway, suggesting that attentional amplification of body-relevant sensory signals may drive the mismatch between perceived and actual stability (47).

### 3.3 Interim summary

The two mechanistic pathways implicated in PPPD, multisensory integration failure and prediction-actual sensory mismatch, converge on attentional processes, but attention should not be treated as a single sufficient cause. In visually dominant patients, exaggerated attention to visual motion may reinforce visual dependence and prevent flexible sensory re-weighting. In patients with quiet standing, sitting, passive-motion, or active-motion intolerance, attention may instead amplify postural prediction errors, vestibular self-motion signals, interoceptive cues, or the cognitive demand of gait and movement control. The following sections examine evidence for attentional dysfunction as a candidate organizing process across these heterogeneous clinical expressions.

## 4 Attentional dysfunction in PPPD: evidence across domains

Converging evidence from neuroimaging, behavioral paradigms, psychophysical studies, questionnaires, and treatment research supports the hypothesis that attentional dysfunction contributes to PPPD maintenance (11–13, 19–24, 31, 40). The evidence is strongest when attention is framed as a network-level modulatory process that changes how sensory, postural, interoceptive, and threat-related signals are weighted. It is weaker if attention is interpreted as a single lesion, a universal visual mechanism, or a proven final common cause. This section reviews the evidence and its limitations across domains.

### 4.1 Neuroimaging evidence: reconfiguration of attention-related brain networks

**Figure 1:**
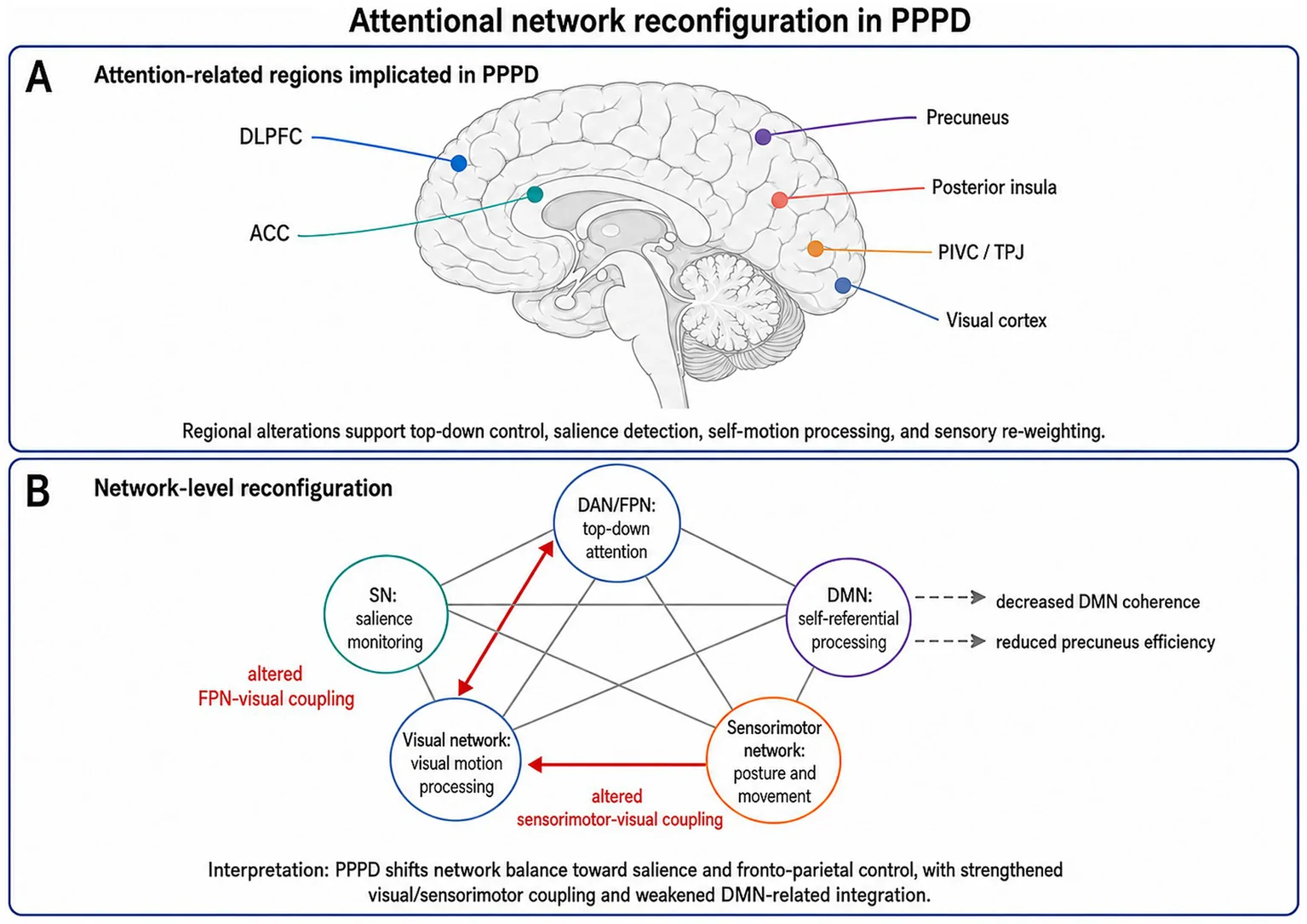
Attentional network reconfiguration in PPPD. **(A)** Schematic localization of regions implicated in PPPD and their main functional relevance in the present model: DLPFC, executive and top-down attentional control; ACC, conflict monitoring and salience-related control; posterior insula and PIVC/TPJ, vestibular, interoceptive, and self-motion integration; precuneus, visuospatial and self-referential processing; visual cortex, visual motion processing. **(B)** Network-level model showing altered frontoparietal-visual coupling and sensorimotor-visual coupling, together with reduced DMN coherence and reduced precuneus efficiency. These labels refer to functional network interactions rather than one-to-one anatomical pathways. This schematic was created by the authors based on the literature synthesized in this review.

Functional and structural neuroimaging studies provide biological evidence of distributed network reconfiguration in PPPD (Figure 1) (12, 13, 19–23, 49). The relevant findings involve both regional changes and altered communication among attention, salience, vestibular, visual, sensorimotor, cerebellar, and default-mode networks.

#### 4.1.1 Structural and functional alterations in attention-related and vestibular-integration regions

Structural MRI studies have identified changes in regions that contribute to top-down control, vestibular-multisensory integration, spatial orientation, and postural-motor calibration. Wurthmann et al. reported decreased gray matter volume in the DLPFC, ACC, temporal cortex, hippocampus, caudate nucleus, precentral gyrus, and cerebellum in PPPD (39). Popp et al. reported cortical alterations in PPV involving insular, parietal, visual, cingulate, premotor, and precuneus regions (38). Nigro et al. found reduced cortical folding in multimodal vestibular regions, including posterior insula, parietal and temporal regions, precuneus, cuneus, and lateral occipital cortex (37). These regions should not be interpreted as an “attention circuit” in isolation. Rather, they contribute different functions: the DLPFC to executive and top-down attentional control, the ACC to conflict monitoring and salience-related control, posterior insular and parietal opercular regions to vestibular and interoceptive processing, PIVC/TPJ regions to self-motion and vestibular integration, the precuneus to visuospatial and self-referential processing, and cerebellar and sensorimotor regions to postural-motor calibration.

Functional MRI studies support this distributed interpretation. Indovina et al. found altered insular and ACC responses during sound-evoked vestibular stimulation in chronic subjective dizziness, a predecessor diagnosis of PPPD (14). Riccelli et al. showed altered insular and occipital responses to simulated vertical self-motion in PPPD (15). Lee et al. reported resting-state functional connectivity changes in PPPD, including altered connectivity between visual, vestibular, and anxiety-related networks (17). von Sohsten Lins et al. reported cerebral responses to emotional stimuli in women with PPPD, supporting involvement of affective-salience processing beyond vestibular cortex alone (36). Yagi et al. found abnormal connectivity among vestibular, visual, somatosensory, and spatial cognitive cortical areas before and after visual stimulation (20). Storm et al. later reported stronger GVS-evoked activation in the multisensory vestibular cortical network, including insular/opercular and cerebellar regions, consistent with sensory-neural amplification rather than a purely attentional or purely visual account (21).

#### 4.1.2 Altered inter-network functional connectivity

Beyond regional abnormalities, PPPD is characterized by altered communication among large-scale networks. Li et al. used independent component analysis and graph theory and reported decreased intra-network connectivity within the default mode network (DMN), enhanced connectivity between the sensorimotor and occipital visual networks, and reduced nodal efficiency involving the precuneus and occipital visual cortex (18). Li et al. subsequently extended this network account by showing abnormal neural circuits with key nodes in occipital visual cortex, precuneus, sensorimotor cortex, multisensory vestibular cortex, and cerebellum, together with less efficient information transmission at both local and global levels (19). Qin et al. reported altered spontaneous brain activity and functional connectivity involving regions responsible for vestibular and postural integration, visual–spatial processing, salience monitoring, motor regulation, and self-referential cognitive processing (23). Liu et al. provided additional resting-state evidence distinguishing PPPD from residual dizziness after BPPV, suggesting that PPPD has functional-network features that are not simply equivalent to persistent peripheral vestibular dysfunction (22).

These findings support a network-level model in which attention-related control systems interact with vestibular, visual, sensorimotor, and salience systems. Case-level evidence involving right parietal surgery and agoraphobic symptoms further illustrates how disruption of multimodal vestibular connectivity may affect spatial threat processing, although this evidence is indirect for PPPD itself (50). Overall, neuroimaging studies do not prove that attention is the sole primary abnormality. Instead, they justify treating attention as a plausible regulatory process that may modulate the precision weighting of sensory signals, symptom vigilance, and the balance between externally directed environmental monitoring and internally directed bodily monitoring.

### 4.2 Behavioral evidence: attentional resource competition and maladaptive control

**Figure 2:**
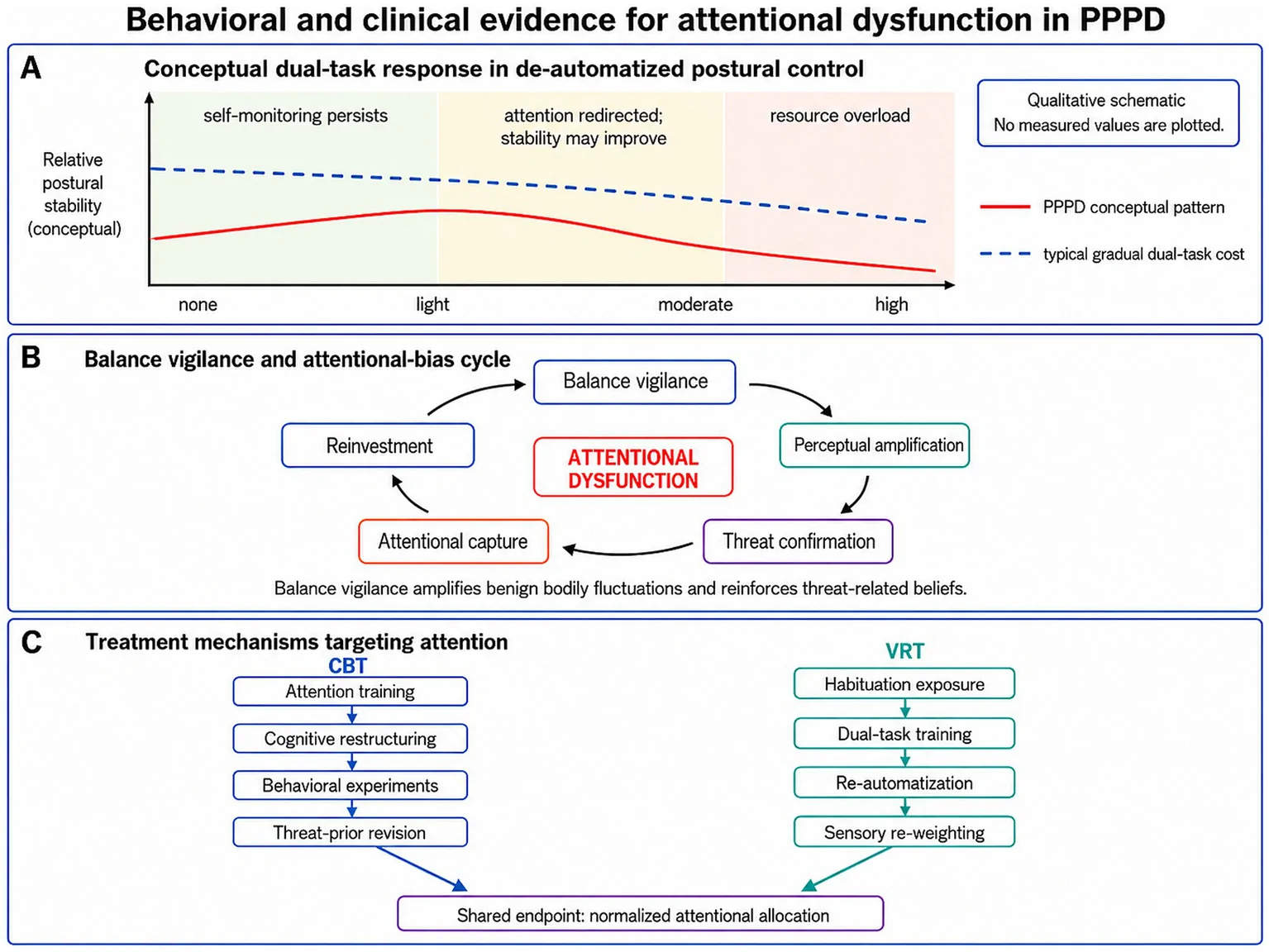
Behavioral and clinical evidence for attentional dysfunction in PPPD. **(A)** Conceptual dual-task response pattern illustrating de-automatized postural control. The curves are qualitative schematics rather than measured values: Light cognitive load may improve stability by reducing maladaptive self-monitoring, whereas higher load may worsen stability as attentional resources are depleted. **(B)** Balance-vigilance and attentional-bias cycle linking balance vigilance, perceptual amplification, threat confirmation, attentional capture, and reinvestment. **(C)** Cognitive behavioral therapy (CBT) and vestibular rehabilitation therapy (VRT) target attention through complementary mechanisms and converge on more flexible attentional allocation. This schematic was created by the authors based on the literature synthesized in this review.

Behavioral studies using posturography, gait analysis, visual exploration, and dual-task paradigms provide a more specific route for linking attention to PPPD mechanisms (Figure 2A). The key issue is not simply that patients perform worse when distracted. Rather, the abnormality appears to be a loss of automaticity, in which posture and gait require conscious monitoring that would normally be unnecessary.

Several PPV studies, now understood as directly relevant to PPPD’s historical phenotype, support this interpretation. Krafczyk et al. found increased high-frequency body sway in PPV, a pattern interpreted as a stiffened strategy linked to conscious control and co-contraction rather than frank postural collapse (26). Wuehr et al. showed abnormal coordination between open-loop and closed-loop postural control, consistent with inappropriate use of sensory feedback during quiet stance (25). Schniepp et al. found cautious gait alterations in PPV and showed that attention and visual input influenced gait performance, supporting increased attentional control and visual reliance during locomotion (27). Penkava et al. extended this evidence to oculomotor behavior by showing altered spontaneous visual exploration during walking in patients with PPV (28).

This interpretation is also supported by broader motor-control evidence. Movement reinvestment describes the tendency to consciously monitor and control movements that are normally automatic (51). Experimental work indicates that conscious processing of balance can distort perceived instability, especially under perceived postural threat or limited balance capacity (50, 52). These findings provide a plausible behavioral bridge between general motor-control theory and PPPD, in which persistent self-monitoring may interfere with automatic postural regulation.

These studies help explain why dual-task effects are important but must be interpreted carefully. A light cognitive task may improve stability in some patients by diverting attention away from maladaptive self-monitoring, whereas a more demanding task may worsen performance once attentional resources are exhausted (11, 25, 27). This non-linear pattern differs from a simple generic dual-task cost. It suggests that excessive self-monitoring can itself destabilize automatic postural control, but also that patients have limited spare capacity when posture and gait already consume cognitive resources.

Recent PPPD patient studies add direct posturographic and perceptual evidence. Söhsten Lins et al. found abnormal Sensory Organization Test performance across multiple sensory conditions (30). Helmchen et al. showed that perceived sway can exceed objectively measured sway, supporting a mismatch between body-state perception and actual postural motion (47). Storm et al. reported altered visual and vestibular motion perception, including false-alarm motion perceptions in sham conditions (46). More recent visual-vestibular stimulation work showed that PPPD patients can report stronger egomotion than controls even in supine or standing positions, and that postural sway responses depend on surface, vestibular stimulation, and stimulus context rather than visual stimulation alone (31, 40). Together, these findings suggest that attentional gain may operate across postural, vestibular, visual, and interoceptive signals, not only through visual dependence.

### 4.3 Clinical evidence: hypervigilance, attentional bias, and treatment response

Clinically, PPPD is characterized in many patients by heightened interoceptive and postural attention, often termed “balance vigilance (Figure 2B)” Balance vigilance refers to persistent monitoring of postural sensations and perceived instability (53). In a self-reinforcing cycle, heightened monitoring can amplify minor physiological fluctuations in balance, heartbeat, respiration, vestibular sensations, or visually induced self-motion. These sensations may then be interpreted as evidence of instability, motivating further vigilance and reinforcing threat-related beliefs about bodily vulnerability (3, 53).

The Balance Vigilance Questionnaire (Balance-VQ) was developed to capture this construct, and validation data support elevated balance-related attention in patients with PPPD and related chronic dizziness disorders (53). This construct is clinically useful because it distinguishes adaptive attention to balance during genuinely difficult conditions from rigid, persistent monitoring that becomes decoupled from objective postural threat. It also helps explain why patients may feel unstable during apparently safe conditions, including quiet standing, sitting, or environments without an obvious vestibular challenge.

Beyond generalized hypervigilance, PPPD patients may show attentional bias toward dizziness-related and motion-related cues. Threat-related attentional bias is well established in anxiety research (24), and PPPD commonly coexists with anxiety, neurotic traits, and threat-related interpretations of bodily sensations (5, 11). However, the presence of anxiety does not mean that PPPD is reducible to anxiety. The clinically relevant point is that attentional bias may become linked to balance, motion, and bodily orientation cues, thereby sustaining dizziness even when peripheral vestibular function is normal or compensated.

Treatment evidence is compatible with this interpretation but does not prove it. CBT for PPPD targets catastrophic interpretations, avoidance, safety behaviors, and self-monitoring; meta-analytic evidence suggests benefit, particularly when CBT is added to conventional therapy, although larger and better standardized trials would strengthen the evidence base (13, 54). Vestibular rehabilitation therapy (VRT) may reduce dizziness handicap through habituation, graded exposure, sensory re-weighting, gaze stabilization, and restoration of automatic movement control (55, 56). Dual-task training can be interpreted as one component of attentional retraining because it encourages patients to move without excessive conscious monitoring. However, treatment response is heterogeneous, and recent original clinical studies emphasize subjective-objective dissociation, multimodal treatment response, and the potential value of optimizing rehabilitation by phenotype and symptom duration (6, 56).

### 4.4 Clinical heterogeneity and symptom subtypes

One of the most important limitations of a purely visual-dependence account is that PPPD is clinically heterogeneous. The diagnostic criteria require exacerbation by upright posture, motion, and visual stimulation, but these triggers do not affect all patients to the same degree (1). Some patients are dominated by visually induced dizziness, whereas others are primarily limited by walking, passive motion, quiet standing, or even sitting. A review model that equates PPPD with visual dependence therefore risks missing clinically important subgroups.

Questionnaire studies provide an empirical basis for this heterogeneity. The Niigata PPPD Questionnaire (NPQ) was developed to assess the severity of PPPD symptoms across three exacerbating domains: upright posture or walking, movement, and visual stimulation (41). Yagi et al. later used NPQ factor and cluster analyses to identify PPPD subtypes, showing that visual factors were common but that PPPD was not a uniform visual disorder (42). The Athens-Lubeck Questionnaire (ALQ) further extended this approach by using an 8-item instrument to distinguish visual intolerance, intolerance to quiet standing or sitting, passive motion intolerance, and active motion intolerance (43). In that prospective cohort, approximately two-thirds of patients showed a predominant symptom subtype, with active motion intolerance being the most common phenotype (43).

Recent clinical data distinguishing primary from secondary PPPD also support this heterogeneity, showing that secondary PPPD can carry a different burden of concomitant symptoms, particularly nausea and vomiting in male patients (57). These data require a modification of the attention model. Attention may be a cross-domain regulatory process rather than a mechanism tied exclusively to visual dependence. In visual-intolerance phenotypes, attention may increase the weighting of visual motion signals as reliable and reinforce visual scanning or avoidance. In quiet standing or sitting intolerance, attention may amplify interoceptive and postural prediction-error signals even without overt movement. In passive-motion intolerance, attention may heighten vestibular self-motion prediction errors. In active-motion intolerance, attention may interfere with the automaticity of gait and head-body coordination. This subtype-sensitive framing leaves open the possibility that distinct or partly overlapping mechanisms underlie different PPPD phenotypes.

### 4.5 Summary

Across neuroimaging, behavioral, psychophysical, questionnaire, and treatment domains, evidence supports attentional dysfunction as a candidate organizing process in PPPD. The strongest version of this hypothesis is not that attention alone causes PPPD, but that abnormal attentional allocation may regulate how visual, vestibular, somatosensory, interoceptive, postural, and threat-related signals are weighted. This interpretation accounts for visual dependence where it is present, while also accommodating non-visual phenotypes and symptoms during sitting, passive motion, or perceived egomotion without overt body motion.

## 5 A revised integrative model: attention as a candidate organizing mechanism

**Figure 3:**
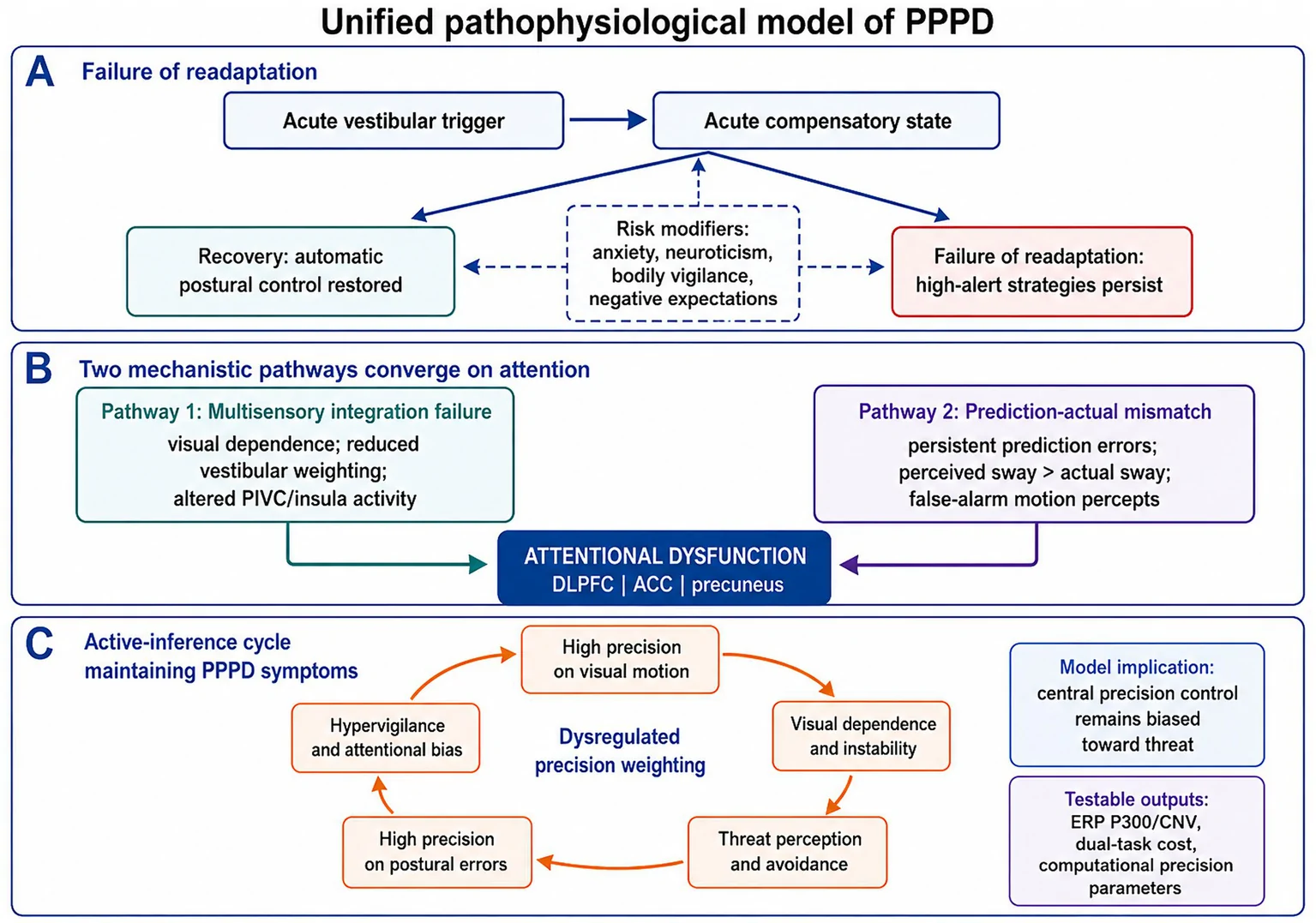
Unified pathophysiological model of persistent postural-perceptual dizziness (PPPD). **(A)** After a vestibular, migraine-related, medical, psychological, or perceived-instability trigger, most individuals return to flexible automatic control, whereas vulnerable individuals may maintain high-alert postural or perceptual strategies. **(B)** Multisensory integration failure and prediction-actual mismatch may converge on attentional dysregulation involving executive-control, salience, vestibular, visual, sensorimotor, and default-mode networks. **(C)** Within an active-inference framework, dysregulated precision weighting may maintain a self-reinforcing cycle linking visual dependence, amplified postural or self-motion prediction errors, threat perception, avoidance, and hypervigilance. This schematic was created by the authors based on the literature synthesized in this review.

The active inference framework provides a computational language for integrating multisensory re-weighting, prediction-actual mismatch, symptom vigilance, spatial cognition, and clinical heterogeneity (Figure 3) (33, 48, 58). In this framework, precision denotes the estimated reliability of sensory signals and prediction errors, which helps determine which signals dominate perceptual inference. Attention is one mechanism by which precision is dynamically regulated (48). Excessive visual dependence can therefore be reframed as abnormally high precision weighting of visual motion signals relative to vestibular signals. Perceived instability despite limited objective sway can be reframed as abnormally high precision weighting of postural or interoceptive prediction errors (11, 31, 40, 46, 47).

This model also explains how PPPD symptoms can arise without a preceding vestibular disorder or without overt body motion. A precipitating event need not be a peripheral vestibular lesion. It may be a medical illness, psychological distress, migraine-related sensory instability, panic-related bodily alarm, orthostatic symptoms, prolonged imbalance, or a perceived threat to bodily orientation (1, 5). Once a high-alert perceptual state is established, attention may continue to enhance precision weighting for self-motion, postural, visual, vestibular, or interoceptive prediction errors. In sitting or supine conditions, the dominant signal may not be body sway itself, but internally generated sensations, visually induced self-motion, vestibular noise, or mismatch between expected and perceived bodily stability (31, 40).

The neuroimaging evidence is compatible with this account. The DLPFC contributes to top-down attentional control, the ACC to conflict and salience-related control, the posterior insula and parietal operculum to vestibular and interoceptive processing, the PIVC/TPJ to vestibular self-motion integration, the precuneus to visuospatial and self-referential processing, and cerebellar and sensorimotor regions to postural-motor calibration (14, 15, 18–23, 37, 59). Altered frontoparietal-visual, sensorimotor-visual, vestibular-cerebellar, salience, and DMN interactions may therefore bias the system toward persistent monitoring and inefficient updating rather than flexible readaptation.

The chronicity of PPPD can be understood as a self-reinforcing cycle. A destabilizing event or perceived threat produces an adaptive shift toward conscious monitoring and increased precision weighting of sensory signals. In vulnerable individuals, including those with high neuroticism, elevated bodily vigilance, anxiety, migraine-related sensory sensitivity, or negative expectations, this shift may become self-sustaining (5, 11, 24). Heightened attention amplifies sensory gain, stronger prediction errors reinforce threat priors, and avoidance or stiffened movement confirms the expectation that ordinary motion is unsafe. The resulting cycle may persist after peripheral vestibular recovery because the maintaining abnormality lies in central regulation of precision weighting, threat appraisal, and attentional allocation rather than in a fixed peripheral deficit.

This revised model is intentionally provisional. It does not claim that attention is the single cause of PPPD. Instead, it proposes that attentional dysregulation may be one candidate organizing mechanism that links several pathways and differs in expression across symptom subtypes.

## 6 Future directions

The revised model suggests several potential priorities for future research, particularly because recent systematic reviews and patient studies continue to find heterogeneity in study design, intervention content, outcome measures, and clinical phenotype (12, 13, 49, 54–56). First, future studies could stratify PPPD patients by symptom phenotype using instruments such as the NPQ and ALQ, rather than treating visual dependence as representative of the whole disorder (41–43). Second, dual-task paradigms may be designed to distinguish light attentional distraction, which may reduce maladaptive self-monitoring, from higher cognitive load, which may expose limited attentional reserve. Third, neuroimaging studies could predefine region-specific and network-specific hypotheses rather than attributing broad functions such as “attention” to all altered regions. Fourth, high-temporal-resolution approaches such as ERP recording may help characterize the dynamics of attentional allocation that fMRI cannot capture. Candidate biomarkers may include dual-task cost, visual exploration measures, postural sway frequency or complexity, ERP components such as P300 and contingent negative variation, auditory middle-latency habituation measures, and computationally estimated precision parameters from active inference models (60–62).

Therapeutically, attention-informed interventions could be tested in phenotype-stratified trials (Figure 2C). CBT may be most relevant when threat priors, avoidance, and balance vigilance are prominent (13, 54). VRT may be most relevant when habituation, sensory re-weighting, gaze stabilization, and restoration of automatic movement control are central treatment goals (55, 56). Patients dominated by quiet standing or sitting intolerance, passive motion intolerance, or active motion intolerance may benefit from different therapeutic emphases than patients dominated by visual intolerance. Emerging approaches such as attention bias modification, graded virtual-reality-based visual motion exposure, and neuromodulation of the DLPFC could be evaluated with mechanistically informed outcomes (63). In the longer term, mechanism-based subtyping could guide treatment selection toward mechanism-guided rehabilitation for PPPD.

## 7 Conclusion

PPPD is a common and disabling functional neuro-otological disorder in which symptoms may persist after, or sometimes without, a clearly documented peripheral vestibular trigger. This review synthesizes neuroimaging, behavioral, psychophysical, questionnaire, and treatment evidence to propose attentional dysfunction as a candidate organizing mechanism rather than a single established cause. Within an active inference framework, attentional dysregulation may bias precision weighting toward visual motion, vestibular self-motion, somatosensory, interoceptive, or postural prediction-error signals depending on the patient’s phenotype. This account explains why visual dependence is important in many patients but insufficient to explain the whole PPPD spectrum. It also accommodates symptoms during quiet standing, sitting, passive motion, and perceived egomotion without overt body motion. The model remains hypothesis-generating and requires direct testing in prospective, phenotype-stratified, mechanism-based studies. Its main value is to connect attention, prediction, sensory integration, and clinical heterogeneity into a framework that can generate testable biomarkers and targeted rehabilitation strategies.
